# Anti-inflammation and Anti-insulin Resistance of a Compound from Vietnamese *Clerodendrum chinense* Leaves

**DOI:** 10.5812/ijpr-169136

**Published:** 2026-04-20

**Authors:** Thu Thi Xuan Nguyen, Cuong Trinh Tat

**Affiliations:** 1 Institute of Biotechnology, Hue University, Hue, Vietnam; 2 National Key Laboratory of Enzyme and Protein Technology, University of Science, Vietnam National University (VNU), Hanoi, Vietnam

**Keywords:** Anti-inflammatory Activity, Anti-insulin Resistance, *Clerodendrum chinense*, Diabetes, Hispidulin, NADPH Oxidase, Reactive Oxygen Species.

## Abstract

**Background:**

*Clerodendrum chinense* has been reported to possess several important biological activities. However, the anti-inflammation and insulin-related properties of isolated compounds from this plant have not yet been investigated.

**Objectives:**

This study focuses on extracting compounds and evaluating improvements in insulin resistance through the anti-inflammatory potential and inhibition of reactive oxygen species (ROS) of hispidulin from Vietnamese *C. chinense* leaves.

**Methods:**

Ethanol was used to separate the compounds from the leaves of Vietnamese *C. chinense*. 1D, 2D-NMR, and ESI-MS spectra were used to analyze the structural identity of the isolated compounds. Compound 1 (hispidulin) was evaluated for anti-inflammation and ROS in Raw 264.7 cells: Anti-inflammatory cytokine inhibition ability was analyzed by the ELISA method; Western blotting was used for the expression levels of phosphorylated ERK1/2, p38, p47phox, and GLUT4. ROS was assessed by immunofluorescence; a luminometer device was used to measure NADPH oxidase activity. The ability to absorb glucose and counteract insulin resistance was evaluated in 3T3-L1 cells via insulin-induced glucose uptake using immunofluorescence dye.

**Results:**

One compound isolated from the leaves of Vietnamese *C. chinense* was identified as hispidulin. Hispidulin at a concentration of 50 μg/mL significantly inhibited tumor necrosis factor (TNF)-α, interleukin (IL)-6, and IL-8 in LPS-induced macrophages (P < 0.001). Moreover, in LPS-stimulated macrophages, the phosphorylation of p38 and ERK1/2, NADPH oxidase activity, as well as the phosphorylation of p47phox, were reduced at a concentration of 50 μg/mL of hispidulin (P < 0.001). More importantly, 3T3-L1 cells with inhibited insulin activity were able to regain glucose uptake and insulin responsiveness through GLUT4 expression when treated with 50 µg/mL of hispidulin (P < 0.001).

**Conclusions:**

The data confirmed the presence of hispidulin in the chemical composition of Vietnamese *C. chinense* leaves. The results indicated that hispidulin inhibited LPS- and ROS-mediated inflammation in macrophages. More importantly, the data have shown that hispidulin enhances glucose uptake and improves insulin activity in 3T3-L1 cells.

## 1. Background

Insulin resistance and chronic inflammation have long been regarded as cornerstones of the etiology of type 2 diabetes mellitus (T2DM) (1). The simultaneous presence of insulin resistance and inflammation may lead to a series of adverse health outcomes, increasing the risk of hyperglycemia, cardiovascular conditions, dyslipidemia, hypertension, and other metabolic disorders (2). Insulin resistance is closely related to the chronic inflammation process through the sustained release of pro-inflammatory cytokines such as tumor necrosis factor (TNF)-α and the aberrant activation of the NF-κB transcription factor (3, 4).

Reactive oxygen species (ROS) in the body can be generated from many pathways, such as nicotinamide adenine dinucleotide phosphate (NADPH) oxidase and cyclooxygenase (COX) (5). Seriously, ROS can be associated with several diseases, including diabetes (6). When blood sugar increases in diabetes, it leads to the formation of advanced glycation end products (AGEs). This process can damage the structure and function of cells (7). The process of creating these proteins leads to dysfunction in insulin signaling and directly affects glucose metabolism into energy (8, 9). Therefore, currently, some compounds have also focused on inhibiting the activity of ROS to study diabetes, such as diosgenin (10), rutin (11), and compound GKT137831 (12).

In modern medicine, chemical therapies and pharmaceuticals are used to manage chronic inflammation and insulin resistance. However, these therapies are associated with adverse effects and risks, particularly with long-term use. In contrast, plant-derived compounds can limit side effects. Therefore, they have attracted significant attention as alternative therapeutic agents. These natural products, which include flavonoids, polyphenols, and terpenoids, are known for their numerous biological activities (3).

*Clerodendrum chinense*, commonly known as glory bower, is a medicinal herb widely distributed in East and Southeast Asia (13). In Vietnam, it is known as "bach dong nu". This flowering ornamental plant, belonging to the Lamiaceae family, is traditionally used locally for general therapeutic purposes, as an analgesic, and to treat conditions such as arthritis, rheumatism, edema, swelling, and gout. Phytochemical investigations have revealed an array of bioactive compounds within *C. chinense*, such as terpenoids, saponins, carbohydrates, glycosides, alkaloids, flavonoids, and phenolic compounds, which are associated with its pharmacological activities (14). Extracts and compounds isolated from *C. chinense* have many important activities, including anticancer, anti-inflammatory, and antibacterial effects (15, 16). Hispidulin, a compound abundant in the *Clerodendrum* genus, has been shown to possess anti-inflammatory, neuroprotective, anticancer, and antidiabetic properties (16).

## 2. Objectives

The anti-inflammatory, ROS inhibition, and hypoglycemic activities of the phytochemical constituents in *C. chinense* leaf extract have not yet been fully clarified. Therefore, this study focuses on isolating and determining the anti-inflammatory and antidiabetic potential of hispidulin isolated from the ethanol extract of *C. chinense* collected in Hanoi Province, Vietnam.

## 3. Methods

### 3.1. Plant Material

In the spring (March 2025), the species *C. chinense* was selected in Ba Vi District, Hanoi, Vietnam (21°03′54″N, 105°20′45″E). Hoang Tan Quang identified the plant at the Institute of Biotechnology, Hue University. A voucher specimen (CC1) has been deposited at the Laboratory of Gene Technology-Institute of Biotechnology, Hue University. The harvested leaves were subsequently dried at 60°C to a constant weight and ground into a fine powder.

### 3.2. Chemicals

Merk supplied lipopolysaccharide, dexamethasone, dimethyl sulfoxide, rosiglitazone maleate, and dihydroethidium; antibodies against phospho-(Ser345)-p47phox, glucose transporter 4 (GLUT4), GAPDH (Glyceraldehyde-3-phosphate dehydrogenase), and TLC silica gel 60 F254 sheets. Cell Signalling Technology (Beverly, MA) provided antibodies against phospho-ERK1/2 and phospho-p38. Antibody against p47phox was obtained from Thermo fisher scientific. The TNF-α, interleukin (IL)-6, and interleukin (IL)-8 ELISA assessment kits were purchased from BD Pharmingen (Franklin Lakes, NJ).

### 3.3. Analysis of the Structure

The Bruker Avance III 600 instrument was used for NMR spectrum analysis in methanol-d4 (CD3OD) solvent with tetramethylsilane. ESI-MS spectra were analyzed on an Aglient 1100 LC/MS spectrometer. Melting point was measured on Stuart SMP20, UK, UV spectrum was measured on SP-UV1100 UV/Vis meter, Dlab, USA. Optical rotation was measured on WXG-4, China.

### 3.4. Extraction and Isolation

Dry powder sample of *C. chinense* (3.0 kg) were extracted with ethanol three times (3x 10L) for 72 hours at room temperature, then concentrated under reduced pressure to give ethanol residue (BDN, 125 g). This residue was suspended in water and then partitioned with n-hexane (BH, 266 g), ethyl acetate (BE, 46,8 g), and butanol (PB, 8,6 g) layers after removal of the solvents in vacuo. The BE layer (46,8 g) was chromatographed on a silica gel column (id: 3.5x 90 cm, 450 silica gel) and eluted with gradient elution n-hexane-acetone (8: 1- 0:1, v/v) to obtain five sub-fractions, BE1 (2.3 g), BE2 (4.6 g), BE3 (8.7 g), BE4 (9.4 g), and BE5 (1.8 g). The BE4 (9,4 g) fraction was chromatographed on an RP-18 (YMC) column (id: 2.5x 80 cm, 120 g RP-C18) eluting with the mobile phase acetone–water (2.75:2, v/v) to yield compound 1 (BDN1, 26.5 mg).

### 3.5. Cell Culture

Raw 264.7 and 3T3-L1 cells were cultured according to the supplier's instructions. Cells were cultured under the following conditions: 95% humidity and 5% CO_2_ at 37°C.

### 3.6. Hispidulin Cytotoxicity Analysis

The toxicity of hispidulin on the survival of Raw 264.7 and 3T3-L1 was assessed. Hispidulin concentrations were incubated in wells at a density of 1×10⁵ cells. Cell Counting Kit-8 (CCK-8, Japan) was used to assess cell survival.

### 3.7. Enzyme-Linked Immunosorbent Assay

LPS-induced proinflammatory cytokines in macrophages were assessed by ELISA. LPS (1 µg/mL) was added for 18 hours after cell pretreatment with hispidulin concentrations for 45 min.

### 3.8. Western Blotting Analysis

For the evaluation of the phosphorylation process of p38, ERK1/2, and p47phox, LPS (1 µg/mL) was treated with Raw264.7 for 30 minutes after cells had been incubated with hispidulin or DMSO (0.1%) for 45 minutes. Cells were lysed as previously described (17). Fifty µg of protein was added to each well of the SDS-PAGE gel. After electrophoresis, the proteins were transferred into a polyvinylidene fluoride membrane. The following antibodies were added in sequence at a 1:1000 dilution: phospho-ERK1/2, phospho-p38, GAPDH, phospho-(Ser345)-p47phox, and p47phox. Then, the HRP-conjugated anti-rabbit antibody (Cell Signaling Technology) was added. Subsequently, the chemiluminescence method was performed (ECL; Amersham-Pharmacia).

To evaluate the phosphorylation of GLUT4, hispidulin, rosiglitazone maleate, and DMSO (0.1%) were incubated with 3T3-L1 cells for 24 days after the cells were treated with TNF-α (1 ng/mL) for 4 days as previously described (18). The cells were then incubated with insulin at a concentration of 100 nM for 5 minutes. Next, the cells were lysed and 50 µg of protein was transferred to each SDS-PAGE gel well. After electrophoresis, the proteins were transferred into a polyvinylidene fluoride membrane. Antibodies were added to GLUT4 (1-1000) and GAPDH (1-1000), respectively. The secondary antibody used was an anti-rabbit antibody linked to HRP (Cell Signaling Technology). The membranes were developed by chemiluminescence assay (ECL; Amersham-Pharmacia). The density of each band was measured using ImageLab version 4.1 (Bio-Rad) software, which was used to evaluate the density of each band.

### 3.9. Reactive Oxygen Species Analysis

Macrophages were treated with hispidulin or DMSO (0.1%) for 45 minutes (19). LPS was then incubated with the cells. Finally, DHE was added to the cells and incubated for 15 minutes at 37ºC in 5% CO_2_. Fluorescence density was assessed using laser scanning fluorescence microscopy (LSM 510) and Carl Zeiss microscopy (LSM510).

### 3.10. Assessment of NADPH Oxidase Activity

Lucigenin chemiluminescence was used to assess NADPH oxidase in macrophages as described previously (17).

### 3.11. Assessment of Glucose Uptake Induced by Inactivated Insulin

The evaluation of hispidulin's glucose uptake was performed as described (18). Fifty ng/mL of TNF-α was pretreated with 3T3-L1 cells for 24 hours. Cells were washed with PBS before incubation with hispidulin. After 24 hours, PBS was used to wash the cells and further incubation for 3 h at 37°C with low-glucose DMEM. The medium consisted of bovine serum albumin (BSA) 1 mg/mL containing 100 mmol/L 2-[N-(7-nitrobenz-2-oxa-1,3-diazol-4-yl) amino]-2-deoxy-glucose (2-NBDG-glucose) and 100 nmol/L insulin, which were replaced and incubated for 1 hour at 37°C. A Hidex device was used to measure the optical intensity of 2-NBDG-glucose at λex = 485 nm and λem = 535 nm.

### 3.12. Statistical Method

The mean ± is the standard deviation (SD) from data of three independent experiments (n = 3) and analyzed by Student's t test, using ANOVA for multiple comparisons. The results were statistically significant when P < 0.05.

## 4. Result

### 4.1. Structure of Compound 1

**Figure 1.**
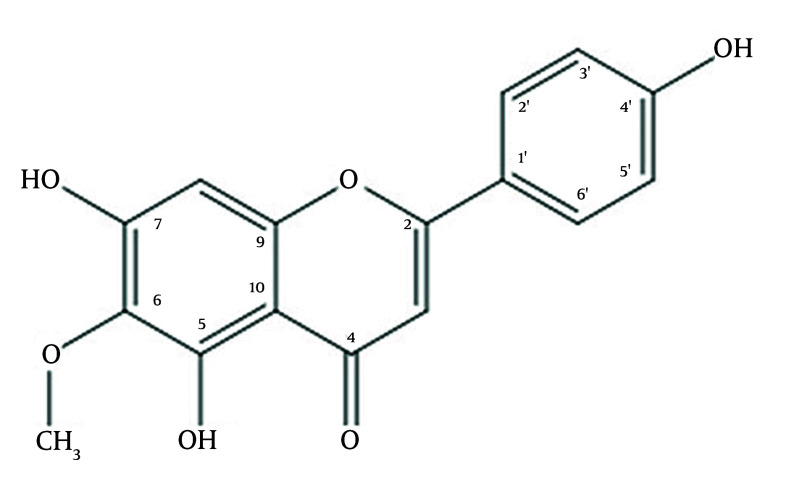
The structure of hispidulin purified from Vietnamese *Clerodendrum chinense* leaves

In the extracted fractions, this study focused on the extraction of hispidulin isolated from the ethyl acetate of *C. chinense* leaves. Based on 1D NMR, 2D NMR (Figure S4 and Figure S5), MS spectra, and comparison with spectral data (20), the chemical structure of compound 1 (Figure 1) was identified as hispidulin.

Hispidulin: Pale yellow needle-shaped crystals; mp. 291℃ (dec); [α]D20 +60° (c 0.2, MeOH); UV (MeOH) λmax: 275, 230, 208 nm; ESI-MS (Figure S1) m/z [M+H]+ 301.2; 1H-NMR (Figure S2) ((MeOD-d4, 600 MHz, δ: ppm) δH 6.55 (1H, s, H-3), 6.56 (1H, s, H-8), 7.81 (1H, d, J= 8.4 Hz, H-2’), 6.92 (1H, d; J=8.4 Hz, H-3’), 6.92 (1H, d; J=8.4 Hz, H-5’), 7.81 (1H; d; J= 8.4 Hz, H-6’), 3.89 (3H; s; 6-OCH3); 13C-NMR (Figure S3) (MeOD-d4, 150 MHz, δ: ppm) δC 166.2 (C-2), 103.3 (C-3), 174.1 (C-4), 153.8 (C-5), 132.7 (C-6), 158.5 (C-7), 95.2 (C-8), 154.5 (C-9), 105.7 (C-10), 123.1 (C-1’), 129.3 (C-2’), 116.9 (C-3’), 162.5 (C-4’), 116.9 (C-5’), 129.3 (C-6’), 60.9 (6-OCH3).

### 4.2. Analysis of the Cytotoxicity of Hispidulin

**Figure 2.**
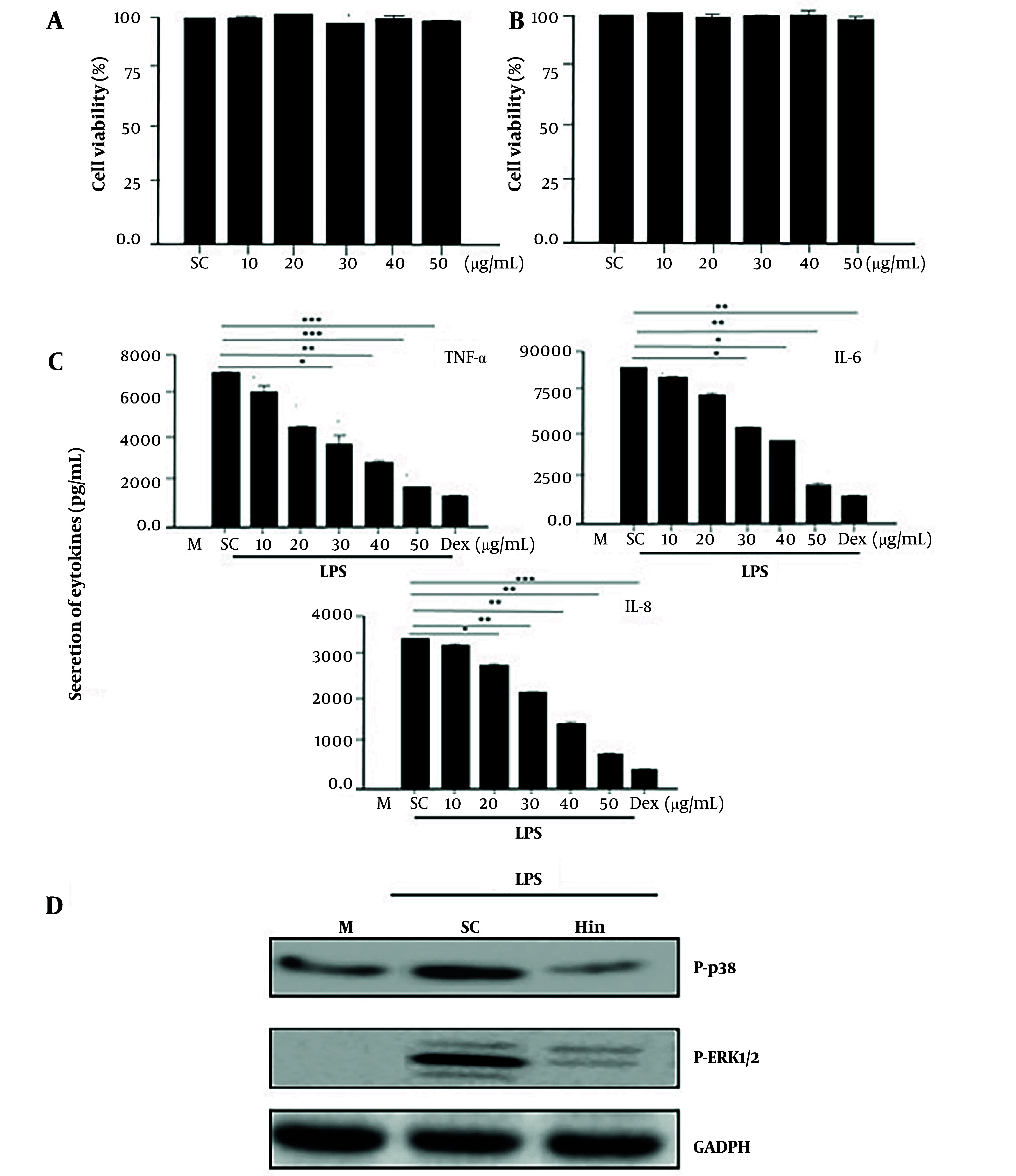
The viability and anti-inflammatory activity of hispidulin in macrophages stimulated by LPS. A, hispidulin concentrations from 10 to 50 µg/mL did not affect the viability of Raw 246.7; B, hispidulin concentrations from 10 to 50 µg/mL did not affect the viability of 3T3-L; C, the secretion of inflammatory cytokines stimulated by LPS was inhibited by hispidulin in macrophages; D, the phosphorylation of ERK1/2 and p38 activated by LPS was inhibited by hispidulin in macrophages, LPS (1µg/mL) was incubated for 30 minutes after cells were pre-treated with either the control solvent or hispidulin (50 µg/mL) for 45 minutes. The experiment was repeated five times (n = 5). LPS, lipopolysaccharide; M, medium; Hin, hispidulin; SC, solvent control (0.1% DMSO). *** P < 0.001 is a significant difference.

**Figure 3.**
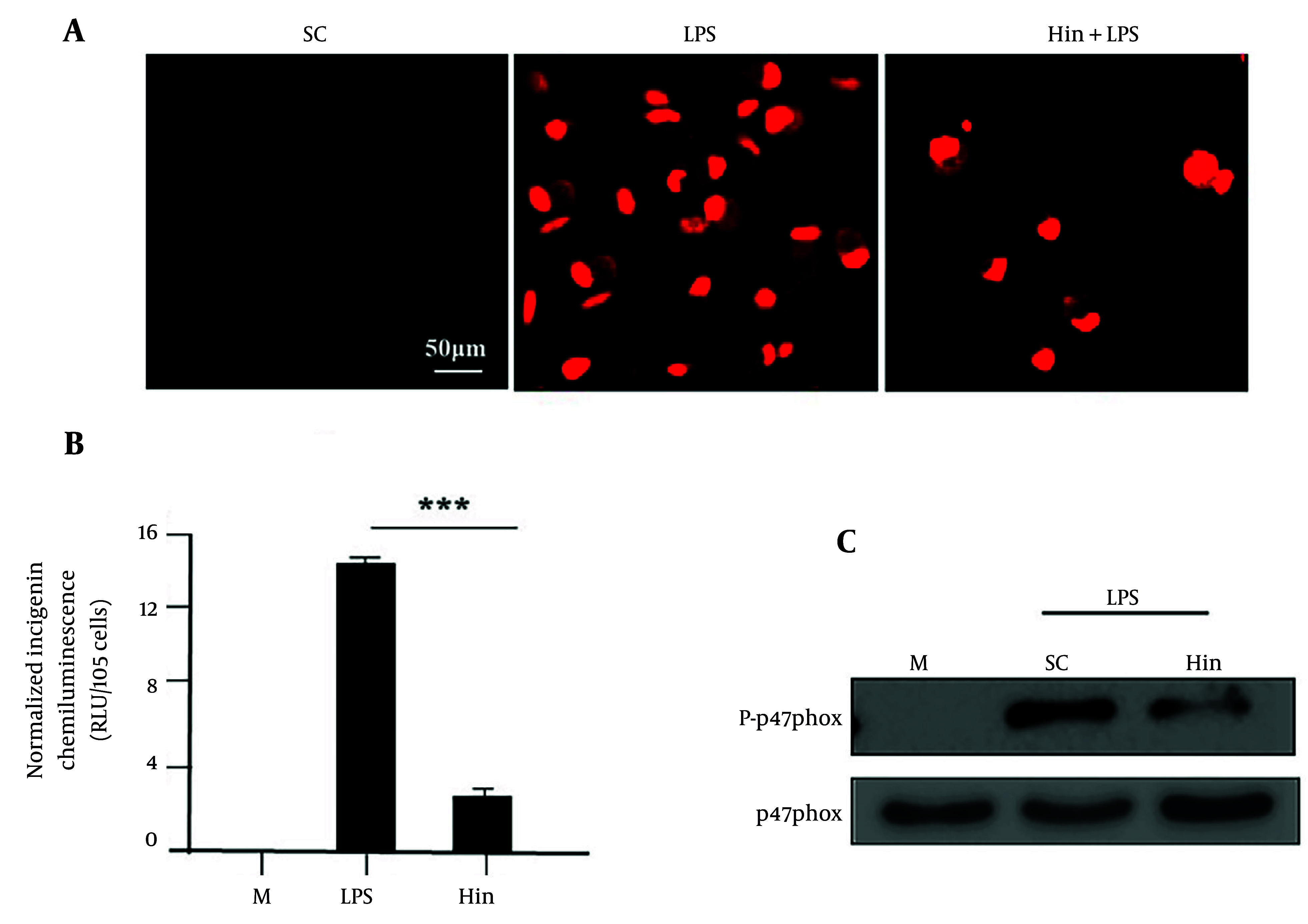
The production of reactive oxygen species (ROS) by LPS is disrupted by hispidulin in macrophages. A, ROS in macrophages were inhibited by hispidulin, LPS (1 µg/mL) was added for 30 minutes after cells were pre-treated with hispidulin (50 µg/mL) for 45 minutes; B, the activity of NADPH oxidase induced by LPS was inhibited by hispidulin in macrophages, LPS (1 µg/mL) was incubated for 30 minutes after cells were pre-treated with hispidulin (50 µg/mL) for 45 minutes; C, the phosphorylation of p47phox was inhibited by hispidulin, LPS (1 µg/mL) was added 15 minutes after macrophages were incubated with hispidulin (50 µg/mL) for 45 minutes, the cells were lysed and approximately 50 µg of protein was added to each well, the phosphorylation of p47phox was analyzed by Western blot. The experiment was repeated five times (n=5). LPS, lipopolysaccharide; M, medium; SC, solvent control (0.1% DMSO). *** P < 0.001 is a significant difference.

Hispidulin's cytotoxicity was evaluated as shown in Figure 2. From this result, it was shown that the concentration range from 10 to 50 μg/mL of hispidulin was not capable of causing toxicity to both Raw 264.7 (Figure 2A) and 3T3-L1 (Figure 2B) cells.

### 4.3. Evaluation of LPS-Induced Inflammatory Inhibition

The concentrations of TNF-α, IL-6, and IL-8 in macrophages treated with LPS were reduced upon incubation with hispidulin (50 μg/mL) compared with cells treated with DMSO (0.1%) (P < 0.001). This result showed that the ability of hispidulin to inhibit proinflammatory cytokines is almost equivalent to Dex (dexamethasone, Figure 2C). Furthermore, p38 and ERK1/2 MAPK play a crucial role in various physiological functions, including the activation of cytokines in macrophages (18). Importantly, the results shown in Figure 2D indicated that hispidulin (50 µg/mL) inhibited the phosphorylation of ERK1/2 and p38.

### 4.4. Determination of Reactive Oxygen Species

LPS activates ROS production in macrophages. However, this process was inhibited by hispidulin (50 μg/mL) as shown in Figure 3A. LPS also stimulates NADPH oxidase activity in macrophages. However, Figure 3B showed that hispidulin (50 μg/mL, P < 0.001) significantly reduced NADPH oxidase activity. Figure 3C showed that the phosphorylation of p47phox to activate NADPH oxidase activity was also inhibited by hispidulin (50 μg/mL). These results indicated that hispidulin inhibited p47phox phosphorylation.

### 4.5. Analysis of the Reactivation of Insulin Resistance

**Figure 4.**
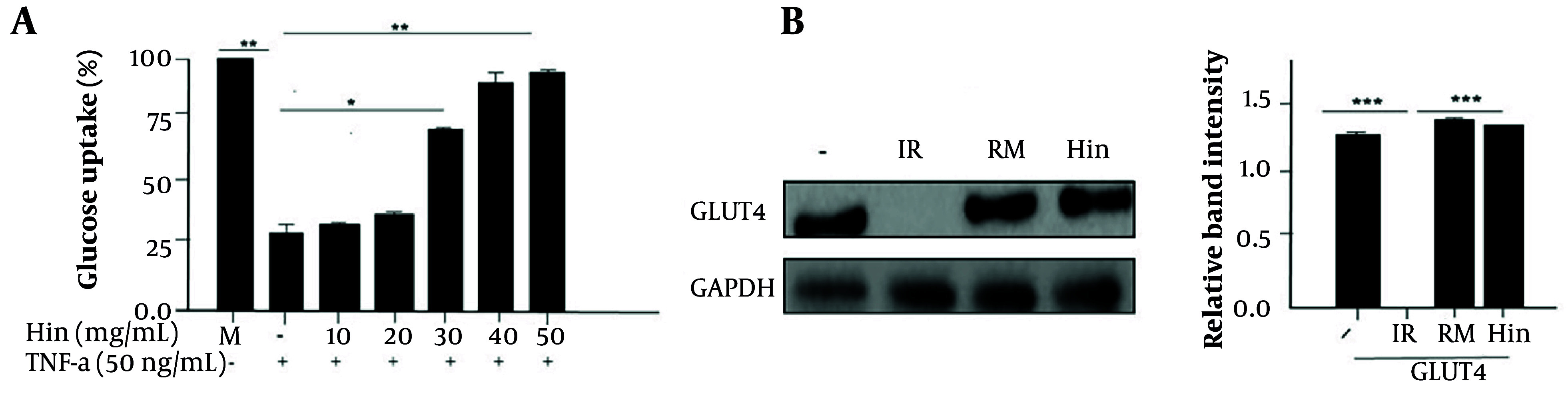
Effect of hispidulin on insulin resistance. A, glucose uptake effect of hispidulin; B, effect of hispidulin on Glucose transporter type 4, the insulin-resistant cells were supplemented with either a control solvent or hispidulin (50 µg/mL) or rosiglitazone maleate (90 μM). The cells were analyzed by Western blotting. -, solvent control (0.1% DMSO); IR, insulin-resistant; RM, rosiglitazone maleate; Hin, hispidulin. The experiment was repeated thrice (n = 5). *** P < 0.001 is a significant difference.

In TNF-α-containing medium, the cells reduced glucose uptake as shown in Figure 4A. However, the results in Figure 4A also showed that hispidulin (50 µg/mL, P < 0.001) increased glucose uptake in 3T3-L1 cells. Importantly, the results in Figure 4B indicated that in the TNF-α-containing medium, GLUT4 phosphorylation was significantly reduced, while this phosphorylation was restored in cells pretreated with hispidulin (50 µg/mL or rosiglitazone maleate, P < 0.001).

## 5. Discussion

*Clerodendrum* is distributed in many parts of the world. Many species of this genus have been classified and applied in traditional medicine to support the treatment of several diseases, for example, antihypertensive, anticancer, antidiarrheal, liver protective, hypoglycemic, and hypolipidemic (13). Species belonging to the genus *Clerodendrum* contain a wide variety of compounds including monoterpenes and derivatives, sesquiterpenes, diterpenoids, triterpenoids, flavonoids and flavonoid glycosides, phenylethanoid glycosides, steroids and steroid glycosides, cyclohexylethanoides, anthraquinones, cyanogenic glycosides, etc. The main and most abundant bioactive compounds are diterpenoids, flavonoids, phenylethanoid glycosides, and steroids (21). In this study, hispidulin was isolated from the leaves of *C. chinense* in Vietnam. This compound has also been isolated from several other species of the genus *Clerodendrum*, such as *Clerodendrum inerme* in South China, India, Southeast Asia, and North Asia and *Clerodendrum petasites* in Thailand, Vietnam, China, India, Malaysia, and Sri Lanka (16). These data also suggest that hispidulin may be present in different genera of *Clerodendrum*. The results in this study showed that TNF-α, IL-6, and IL-8 were inhibited by hispidulin in macrophages when stimulated by LPS. This anti-inflammatory effect is consistent with previously published studies on the activity of hispidulin (22-25). The inflammatory process via ERK1/2 or p38 in macrophages was inhibited by hispidulin, as demonstrated in this study.

Numerous studies have shown that the glucose metabolism pathway in diabetes involves diacylglycerol protein kinase C, which activates NADPH, ultimately producing ROS (6). Therefore, controlling ROS is considered a novel treatment strategy for diabetes (26-28). Reactive oxygen species imbalance can be considered one of the causes of diabetic complications due to hyperglycemia leading to cell death and tissue damage (28).

Currently, oxidative stress is considered a promising treatment approach for diabetic vascular diseases, with inhibition of NADPH oxidase (NOX) being the primary target. Several substances such as rutin (11) and GKT137831 (12) have the ability to control oxidative stress by inhibiting certain molecules in NADPH. Hispidulin was also shown to inhibit NADPH in this study. Previous studies have also indicated that hispidulin possesses numerous bioactive properties that may support the treatment of inflammatory diseases (25) and diabetes (29). Our data are the first to demonstrate the role of hispidulin from *C. chinense* in inhibiting ROS production through inhibition of p47phox phosphorylation in macrophages. In recent years, antioxidant therapy has been studied (5). However, the number of successful clinical trials remains limited. Therefore, exploiting substances of plant origin that have a ROS inhibitory role is still very important to develop more antioxidant-oriented therapeutic drugs. Our data combined with previous studies may suggest hispidulin as a potential anti-inflammatory and anti-oxidant stress inhibitor and support the treatment of diabetes.

Hispidulin has also been reported to have therapeutic potential in streptozotocin-induced diabetes in mice (29), but its ability to protect glucose uptake in adipocytes via TNF-α has not been reported. We demonstrated that low doses of hispidulin (50 μg/mL) improved insulin activity due to the TNF-α-induced intracellular environment. This was demonstrated through the increased glucose uptake in the presence of hispidulin. Furthermore, the insulin receptor substrate (IRS) in cells determines insulin signaling. Activation of this receptor leads to the activation of numerous downstream effects directly related to insulin signaling that regulate intracellular pathways (30). When the receptor binds to insulin on the cell surface, IRS-1 is phosphorylated and activates phosphatidylinositol 3-kinase (PI3-kinase). From here, intracellular signaling leads to cellular responses and activates GLUT receptors (glucose transporters), which increase the amount of glucose absorbed by the cell (31). When insulin resistance occurs, the insulin-GLUT4 pathway is inhibited, which is a major cause of type 2 diabetes as well as several other metabolic diseases. This risk factor still needs further study to identify and improve its effectiveness (32). We have shown in this study that GLUT4 expression in 3T3-L1 adipocytes was significantly increased with the involvement of hispidulin. These results are quite similar to a recent publication that has shown the potential of hispidulin in the treatment of diabetes through the mechanism of mRNA expression of PI3K, AKT (protein kinase B), mTOR (mechanistic target of rapamycin), IRS-1, and GLUT4 while simultaneously reducing GSK-3β (glycogen synthase kinase-3 beta). Therefore, hispidulin increased glucose uptake and insulin signaling via the PI3K/AKT pathway (33). In this study, hispidulin from *C. chinense* was reported to improve insulin resistance in adipocytes via the TNF-α/GLUT4 pathway and inhibit ROS via p47phox. The two publications were evaluated using different models, but both showed that hispidulin has the ability to regulate oxidative stress and increase GLUT4 expression, leading to increased glucose uptake and increased sensitivity to insulin resistance.

### 5.1. Conclusions

In this study, hispidulin was identified and isolated from the leaves of *C. chinense*. The study data suggest that hispidulin has the potential to significantly improve diabetes in an in vitro model. Hispidulin reduced anti-inflammatory cytokines, including TNF-α, by inhibiting the phosphorylation of p38 MAPK and ERK1/2 MAPK. More importantly, hispidulin inhibited ROS and improved GLUT-4 expression, showing anti-insulin resistance effects. Overall, our data suggest the potential of hispidulin to modulate acute inflammation, anti-insulin resistance, and reduce diabetic complications through ROS-dependent pathways.

ijpr-25-1-169136-s001.pdf

## Data Availability

The data presented in this study are uploaded during submission as a supplementary file and are openly available for readers upon request.
