# Supplementary material for: Anti-inflammation and Anti-insulin Resistance of a Compound from Vietnamese Clerodendrum chinense Leaves
Source: Iran J Pharm Res. 2026 Apr 20;25(1):e169136. doi: 10.5812/ijpr-169136 (PMC13187690; doi:10.5812/ijpr-169136)
Supplement: ijpr-25-1-169136-s001.pdf [file ijpr-25-1-169136-s001.pdf]

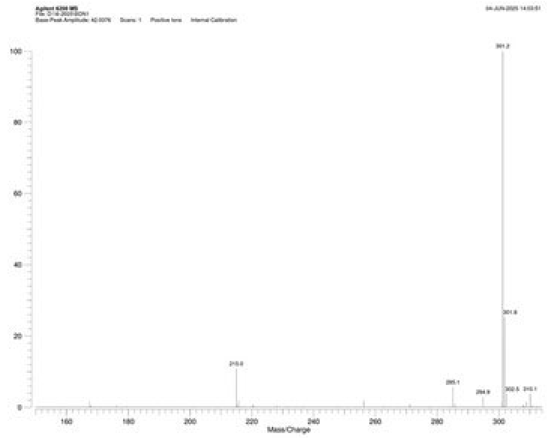

Figure S1. ESI-MS spectrum of compound 1

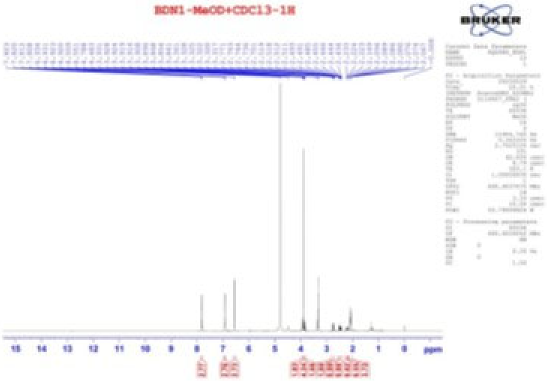

Figure S2. ^{1}H-NMR spectrum of compound 1

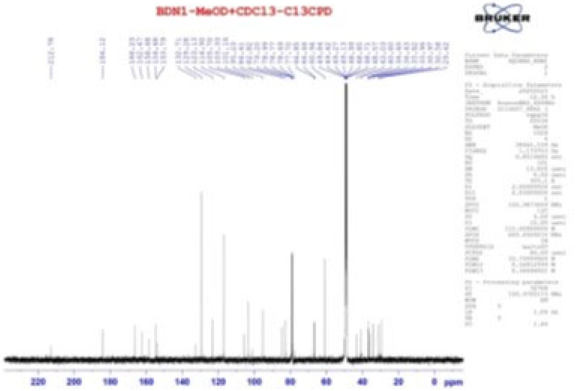

Figure S3. ^{13}C-NMR spectrum of compound 1

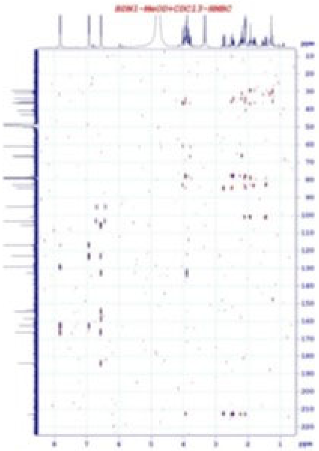

Figure S4. HMBC spectrum of compound 1

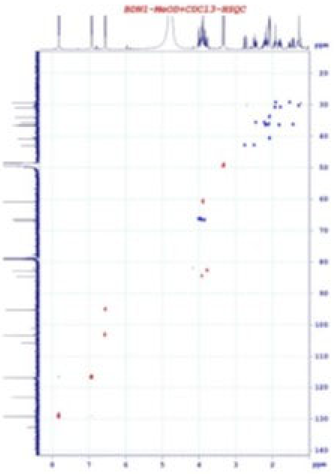

Figure S5. HSQC spectrum of compound 1
